# The Influence of Government Regulation on Farmers’ Green Production Behavior—From the Perspective of the Market Structure

**DOI:** 10.3390/ijerph20010506

**Published:** 2022-12-28

**Authors:** Qiang Huang, Huizhu Wang, Chao Chen

**Affiliations:** College of Economics and Management, Nanjing Agricultural University, Nanjing 210095, China

**Keywords:** government regulation, market structure, green production, intermediary effect

## Abstract

To clarify the factors influencing the green production behavior of peach farmers, this paper uses the survey data of 741 peach farmers in 19 provinces and cities in China, it uses a multiple ordered probit model to empirically analyze the impact of the government regulations on the green production behavior of peach farmers, from the perspective of the market structure. This paper also analyzes its intermediary role in this process, and it analyzes the possible heterogeneity at both the planting scale and the regional level. The results show the following: (1) Government regulation has a positive and significant impact on the green production behavior of peach farmers. Specifically, government supervision and inspection, alongside green subsidies, can positively promote the implementation of green production behavior by peach farmers, but government publicity and training have not played a good role. (2) The market structure plays a partial intermediary role, rather than a complete intermediary role, in the government regulation affecting the green production behavior of peach farmers. (3) The impact of the government regulation on the green production behavior of peach farmers is heterogeneous. Specifically, compared with small farmers, the impact on large-scale farmers is higher; however, the influence of the three methods of government regulation on the green production behavior of peach farmers varies from region to region. Therefore, in order to promote the implementation of green production, the government should introduce appropriate local policies, strongly support new agricultural business entities, draw clear guidelines for the market, and play the role of “night watchman”.

## 1. Introduction

China has high incidence of diseases and insect pests. Thus, pesticides play a great role in protecting the crop yield and ensuring the effective supply. However, for a long time, pesticides have been overused in China. By 2020, the total amount of pesticides used in China was 1.313 million tons, corresponding to 10.3 kg/ha per unit area [1]. In contrast, the pesticide use in Japan is 3.72 kg/ha, that in France is 3.69 kg/ha, and the world average is 2.6 kg/ha (FA0). Therefore, the use of pesticides, per unit area, in China is far higher than the world average. Excessive pesticides not only cause soil compaction, but also lead to serious agricultural pollution in large areas. This hinders the sustainable development of China’s agriculture. The Fifth Plenary Session of the 18th Communist Party of China (CPC) Central Committee put forward the concept of green development; the Ministry of Agriculture began to carry out the work of the “zero growth of using pesticide” in 2015. The report of the 19th National Congress of the CPC called for the strictest ecological and environmental protection measures to foster green ways for development and life, and the No. 1 Document of the CPC Central Committee formulated the strategy to “develop high quality agriculture” in 2018, which once again emphasized a “green style” of agriculture development. In 2021, the No. 1 Central Document of the Central Committee, again emphasized the need to realize the green transformation of the rural production and lifestyle and the continuous reduction of pesticide use. The United Nations Environment Program (UNEP) has appealed to countries to adopt a “green agriculture”, to achieve carbon neutrality by 2030. Therefore, against the current background of double carbon, it is urgent for China to implement a green agricultural production.

The repeated emphasis of the policy shows that China’s agricultural development mode of resource and investment allocation has not changed substantially. That is, government regulation, as one of the important means of government intervention in the economy, has “failed” in the field of microeconomics. As an important micro decision-making subject in agricultural production and operation, the choice of farmers’ behaviors directly affects the quality of the ecological environment and the level of social welfare, and their green production behavior determines the green development of China’s agriculture. The fundamental problem in the farmers’ adoption of a green production mode is mainly reflected in the opposition between the externality of the green production behavior and the rational behavior of the farmer, as an “economic man”. Relevant government regulation must be used to curb the negative externalities, through strict supervision and punishment measures, and to regulate the farmers’ production behaviors to improve the production environment. Meanwhile, farmers’ green production costs can be decreased and their production income increased by various forms of financial subsidies.

The existing research also discusses the impact of the government regulation on the farmers’ green production behaviors [2,3,4]. Government regulation is divided into the command type and incentive type, and previous studies have shown that, as an incentive regulatory policy, government subsidies can close the positive externality income gap of the green production behavior, thus promoting its adoption [5]. Research by Feng et al. (2021) [3] confirmed that the government’s environmental regulatory policies have a strong role in promoting green production in China. However, some scholars hold the opposite view, that although government training and other measures can improve the technical availability of farmers, this requires farmers to invest a certain amount of time and energy, and the training content may not meet their needs, which is likely to cause resentment among farmers [6]. At the same time, some scholars have emphasized that the impact of different types of government regulatory policies is heterogeneous. For example, publicity, education, and technical training can improve the operability of green production technology for farmers, while punishment can increase the standardization of the farmers’ production behaviors [7].

At present, there are 210 million farmers in China with less than 10 mu of arable land. Small-scale decentralized management is still the main form of agricultural management in China [8]. Under this form of management, farmers are less likely to reduce their use of pesticides. Therefore, how to scientifically and reasonably guide and encourage farmers, especially small farmers, to reduce the pesticide application and fully mobilize their enthusiasm and initiative for green production, is the key to promoting the green transformation of the rural production mode and achieving high-quality and sustainable agricultural development. Previous studies have mostly focused on the relationship between the agricultural operation scale and reduction. Most studies have concluded that expanding the scale of operation is conducive to pesticide reduction [9,10]. However, the long-term practice shows that the circulation of agricultural land has not improved the pattern of land fragmentation in China. Therefore, it is a relatively slow process to realize the land scale operation through the agricultural land transfer, thus promoting the pesticide reduction. Currently, the information asymmetry leads to the “the market for lemons”. The market for lemons refers to a market with asymmetric information, that is, compared with the product buyer, the product seller has more information about the product quality in the market. In this case, superior goods are often eliminated, while inferior goods gradually occupy the market and replace the superior goods, thus resulting in a market full of inferior goods. In particular, there exists a phenomenon called “bad money drives out good” in the market of agricultural products. This refers to the problem of the adverse selection caused by the asymmetric information between two parties in the market. This leads to consumption-driven productions, such that farmers are reluctant to participate in green production due to the poor market performance. With the development and improvement of China’s market structure, various industrial organizations can integrate resources, increase information sharing, and reduce operating costs and risks, which has become an important way for China to achieve the transformation of agricultural production mode towards green agriculture [11]. So, as rational economic individuals, farmers prefer to join some organizations. Therefore, in the new stage of agricultural development, which promotes the overall green development of agriculture and leads to the rural revitalization with green development, the development of the market structure can encourage small farmers mainly engaged in household management, to carry out large-scale service management, providing new ideas for reducing the pesticide application.

Some existing research has focused on the potential impact of the market structure on the green production behaviors of farmers. Wang Bing and Huang Dai (2005) [12] believe that the significance of market structure is to facilitate the market entry and exit. Later, many scholars studied the impact of market structures, such as the participation in cooperatives and agricultural leading enterprises, on farmers’ green production behaviors [13,14,15,16]. Most studies have confirmed that the participation in cooperatives has a positive and significant impact on the farmers’ green production behaviors, because participation in cooperatives can improve farmers’ income expectations and ecological awareness, while reducing the capital and risk constraints faced by the farmers [17]. Similarly, the contractual relationship between farmers and the leading enterprises, peach farmers’ associations, and other market organizations, can also significantly promote their adoption of green production technologies. However, some studies have shown that the market structure has no significant impact on the farmers’ green production behaviors [18]. Furthermore, some research has found that the market structure may inhibit the agricultural decrement behavior [19], because the agricultural material dealers are the main channels by which farmers purchase pesticides and obtain pesticide application information. In order to obtain commercial profits, the market organizations may cooperate with the dealers, pushing farmers to purchase and use pesticides beyond the reasonable range of use [20], thus aggravating the unreasonable abuse of pesticides, obstructing the process of green production [13].

As for other factors influencing farmers’ green production behaviors, it is found through sorting through the existing literature that most focus on the endowment and cognition of farmers. In terms of farmers’ endowment, the main focus is on education level, age, social network, planting scale, part-time employment, etc. Most studies show that a farmer who has a higher level of education, is younger, and has a stronger ability to accept new things, is more likely to promote their green production behavior [21]; the expansion of social networks can also promote farmers to adopt green production and social services, thereby reducing the use of chemical fertilizers [22]. An increase in the planting scale can reduce the input of pesticides and fertilizers, encourage farmers to adopt green innovative technologies, and thus attain a green agricultural development [23]; with the deepening of part-time businesses, farmers will no longer rely on the planting income, reducing their adoption of green production technology [24]. However, there also exists contrary research conclusions, suggesting that with the deepening of part-time businesses, farmers will become self-sufficient in their land use, but will pay more attention to the quality of the agricultural output, and promote their adoption of green production technology [25]. In terms of the farmers’ cognition, most studies show that farmers’ green cognition can significantly promote their green production behaviors [26,27,28].

The above research results provide an important reference for this paper, but there are still areas to be improved. First, the existing literature mostly focuses on the direct impact of government regulation or the market structure on green production behavior, but few studies discuss the intermediary effect of the market structure in the process of government regulation affecting farmers’ adoption of green production behaviors, and the mechanism of synergy between the two is not clear. Second, few studies have subdivided government regulation and farmers’ green production behaviors to explore the role of different government regulation methods on different green production behaviors. All of these provide space for further research in this paper. Therefore, under the current background of high-quality agricultural development, this paper uses the survey data of 741 peach farmers across the country to explore the impact mechanism of government regulation and market structure on farmers’ green production behaviors, and investigates the heterogeneity in the different scales and regions. On the one hand, it helps to improve the relevant research system, make marginal contributions, and provide a new perspective for subsequent research; on the other hand, it provides a scientific decision-making basis for the government to consider how to promote green production behaviors.

## 2. Theoretical Analysis and Research Hypothesis

In the field of economics, government regulation was initially defined as the essential rules for industry development and interests, which is the application of the state’s coercive power [29]. Spulber (2003) [30] developed this concept, saying that regulation is a general rule or special behavior that is formulated and implemented by administrative agencies to interfere with the market allocation mechanism or indirectly change the supply and demand decisions of enterprises and consumers. It originates from market failure, taxation, and the government requirement for specific goals [31]. The goal of government regulation is to form an effective market structure to compensate for the market deficiencies, achieve an appropriate scale and monopoly, improve economic efficiency, and increase social welfare. Meanwhile, formulating and implementing regulation is also a bargaining process among various stakeholders.

As for the concept of market structure, from the perspective of neoclassical economics, in terms of social division and labor specialization, the combination of several individual choice structures in the economic environment represents the market structure. This depends on the relative scale of the transaction efficiency and the specialized return growth [32]. The institutional school emphasizes its institutional constraints, such as the property rights, governance structure, and trading rules. The Austrian school believes that it is a process of market cooperation and knowledge discovery. However, in sociology, market structure is a specific interaction between competition and exchange, which depends on the “social network” between power-building competitors. It contains economic, non-economic, market, and government systems [33]. To sum up, from the perspectives of monopoly competition, the division of labor and collaboration, and social network, market structure can be divided into three structures: competition structure, division of labor structure, and social structure. This facilitates the study of the market performance under different market structures.

As an organizational form, such as the market, the government will inevitably improve the market performance by influencing the market structure. First of all, the government, as the main investor and regulator of public affairs, will regulate the market due to various reasons, such as market failure, the seller’s monopoly power, externalities, information cost, and public security. At present, problems, in terms of the quality and safety of the agricultural products market, occur frequently, and due to the existence of market information asymmetry and the time lag effect, farmers seldom use green production behaviors. In order to promote the development of the national economy and society and to ensure people’s health, the government will manage and standardize the production behaviors of farmers. For example, spot checks and tests will be carried out on agricultural products. Unsafe production behaviors will be punished. Meanwhile, related standards of production safety will be formulated for various agricultural products. Studies have confirmed that, in addition to the farmers’ own capacity, external factors, such as the market and government, could affect farmers’ green production behaviors. We therefore believe that government regulation can regulate farmers’ green production behaviors, leading to the following hypothesis:

**H1:**  *Government regulation has a positive impact on farmers’ green production behaviors.*

Different from government regulation, generally speaking, the formation of the market structure is mainly affected by the market environment, objectives and costs among stakeholders, etc. As a result, it is a voluntary market behavior for farmers to join an industrial organization. As rational economic people, farmers will voluntarily join industrial organizations for the maximum benefit when they face government regulations. The standardization and development of industrial organizations, such as cooperatives and leading enterprises, have improved the organization of agriculture, which plays a key role in building a modern agricultural operation system [34]. Industrialization organization can unify and standardize the production behavior of farmers [35]; improving the degree of organization of industry can effectively improve the quality of agricultural products from the source [36]. On the one hand, industrialization organizations standardize and guide the farmers’ production behaviors via contracts. They increase farmers’ incomes by organizing the sale of agricultural products, which causes farmers to have a more positive attitude toward agricultural green production. On the other hand, they provide an exchange platform where members can exchange the implementation of green production with each other. This communication promotes the ordinary farmers’ perceptions of green production. Meanwhile, industrial organizations will provide technical guidance and training to their members, which improves farmers’ understanding of agricultural green production, standardizes their production behaviors, and strengthens farmers’ abilities to control green production. Based on this, the following hypothesis is proposed:

**H2:**  *Market structure has a positive impact on farmers’ green production behaviors; farmers’ green production behaviors vary in different market structures.*

As for government regulation and market structure, they complement each other. Due to the nature of diversification, the market is at risk of failure at the source, such that government intervention is essential to maintain the normal operation of the market. According to the theory of industrial economics, government regulation can adjust and improve unreasonable market structures, and then obtain a good market performance. In other words, an effective market mechanism can only be formed on the basis that the government maintains an effective property rights relationship. This is also the most classic assessment of the relationship between government regulation, market, and property rights. Therefore, it is necessary to study the impact of government regulation on the market structure and performance. The following hypothesis is proposed accordingly:

**H3:**  *Government regulation promotes farmers’ green production behaviors by influencing the market structure.*

Previous studies have shown that farmers’ production behaviors are affected by factors, such as individuals, organizations, markets, and governments, but the logic and path of their influence remain to be further explored. Based on the theory of government regulation and institutional economics, this paper discusses the “blind box” of the influence that government regulation and market structure have on farmers’ green production behaviors.

**Figure 1:**
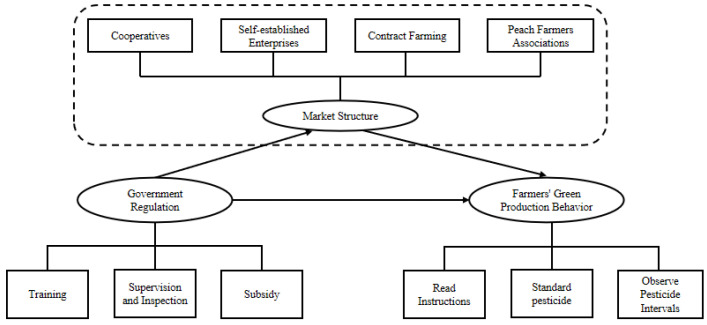
Logical framework.

The logical framework of this paper is shown in Figure 1.

## 3. Research Design

### 3.1. Data Source

The data used in this paper come from the “Peach Garden Production Management Blog” collected by the Industrial Economic Research Office of the National Peach Industry Technology System, during the “13th Five-Year Plan” period (2018–2020), and from special investigations in major production areas in China. The survey covers 17 test stations of the national peach industry technology system located in 19 provinces/municipalities. In the survey, a national major producing area is first chosen, and its two major producing counties are then selected using the stratified sampling method. Finally, major production towns are selected in each county. In the townships, the numbers of peach farmers for household investigation are determined for the township scales of large, medium, and small. The investigators conduct one-to-one interviews with peach farmers. In principle, an adult over 16 years old is interviewed in each household. The survey covers the basic information of farmers, local government policies, market development, market organization mode, and farmers’ green production behaviors. In total, 745 questionnaires per year have been completed. Due to the limited values of the explanatory variables, this study only selected the microscopic data of peach business entities in 2018. Following the removal of the invalid questionnaires that were missing important data, 741 valid samples were obtained, with an efficiency of 99.5%.

### 3.2. Descriptive Statistical Analysis

The following Table 1 is obtained through the descriptive statistics of the samples:

As seen from the above table, the sample farmers have the following characteristics. First, the proportion of males (91.23%) is much higher than that of females (8.77%). This is mainly because in the interviewed households, the head of the household was preferentially recommended as the interviewee. Second, the education level is generally low, with the level of junior high school accounting for 42.91%. This is also in line with the current education status of farmers in China. However, with the development of rural areas, increasing numbers of well-educated people are returning to their hometown and working there. From the sample result, a small proportion of peach farmers have junior college degrees and bachelor degrees, accounting for 12.15% and 3.51%, respectively. Third, the planting period is mainly 0–10 years. Of the respondents, 51.15% have less than 10 years of planting experience, 32.79% of them have been planting for 10–20 years, while only 16.06% of them have more than 20 years of planting experience. Fourth, peach farmers of small-scale farms are still the majority. Peach farmers with less than 50 Mu account for 69.77%, and those with 50–1000 Mu account for a small proportion in each section, with a total proportion of only 29.56%. There are even fewer farmers (only 0.67%) who have more than 1000 Mu. The table demonstrates that in China, current peach planting is still dominated by small-scale farmers.

### 3.3. Model Setting

#### 3.3.1. Ordered Probit Model

In this study, the explained variable “farmers’ green production behaviors” is a classification variable ranging from 1 to 5. The ordered probit model is constructed, as follows:

(1) $${\mathrm{Green}\;\mathrm{production}}_{i}=\alpha_{0}+\alpha_{1}{\mathrm{Goverment}\;\mathrm{regulation}}_{i}+\alpha_{2}{\mathrm{Market}\;\mathrm{strcture}}_{i}+\alpha_{3}Z_{i}+\mu_{i}$$

${Green\;regulation}_{i}$ represents the green production behavior of farmer i; ${Goverment\;regulation}_{i}$ represents government control; ${Market\;strcture}_{i}\;$ represents the market structure; $Z_{i}$ represents a series of control variables, including farmers’ personal characteristics, family operation characteristics, production characteristics, external environment characteristics, and other variables; and $\mu_{i}$ is the random interference term.

#### 3.3.2. Mediating Effect Model

This paper uses the stepwise regression method used by Wen et al. (2004) [37] to test the mediation effect. The mediation effect model is constructed as follows:

(2) $$Green\;production=\;c\cdot Goverment\;regulation+{\;e}_{1}$$

(3) $$Market\;strcture=\;a\cdot Goverment\;regulation+{\;e}_{2}$$

(4) $$Green\;production=\;c^{\prime }\cdot Goverment\;regulation+\;b\cdot Market\;strcture+{\;e}_{3}$$

In Equation (2), c reflects the total effect of government regulation on farmers’ green production behaviors. In Equation (3), a is the influence of government regulation on the market structure of the intermediary variables. Finally, both government regulation and market structure are included in the regression equation to represent their impact on farmers’ green production behaviors. In Equation (4), $c^{\prime }$ and b represent the direct impact of government regulation and the market structure on farmers’ green production behaviors, respectively.

**Figure 2:**
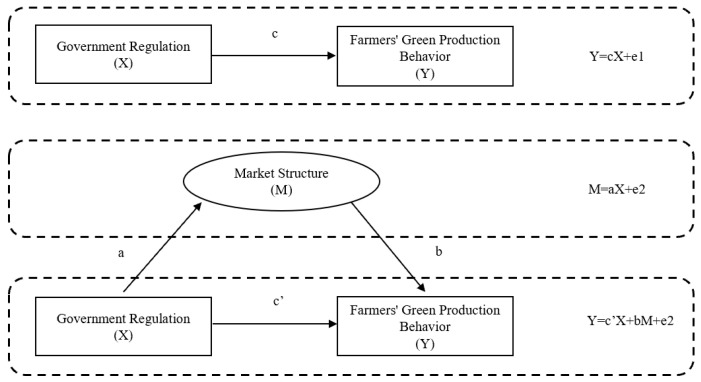
Mediating effect.

The mediating effect is shown in Figure 2.

### 3.4. Variable Selection

#### 3.4.1. Dependent Variables

According to the reports on green economic development and on the definition of farmers’ green production behaviors by the United Nations Environment Program and Li and Zhang (2019) [38], respectively, this paper believes that farmers’ green production behaviors are an agricultural operation mode in which farmers consciously reduce, reuse, and recycle pollution in the process of agricultural production input. In other words, they standardize the pesticide use and pick agricultural products within a safe interval, according to the relevant instructions, such that agricultural products are sold without pesticide residues [39]. Therefore, in order to represent farmers’ green production behaviors, this paper selects several standards, including “whether farmers read the instructions”, “whether pesticides are applied in accordance with the specified dosage instructions”, and “whether the pesticide interval is followed”. These standards all adopt a 5-measure Likert scale.

#### 3.4.2. Independent Variables

This paper takes government regulation and market structure as the core explanatory variables. Government regulation is a series of interventions by the government, in order to change farmers’ production behaviors. Drawing on previous studies [40,41], and according to the needs of this study, government regulation in this paper mainly refers to the guidance regulation, incentive regulation, and constraint regulation that peach farmers are subject to during pesticide spraying, so we use the following three indicators to measure government regulation: “whether the government has conducted publicity and training on pesticide use during planting”, “whether the government has conducted the supervision and inspection of pesticide use during planting”, and “whether the government has provided biological pesticide subsidies”.

Market structure refers to an organization mode that farmers voluntarily join. The preliminary investigation found that the main organizational models chosen by farmers are cooperatives, contract farming, joining a peach farmers’ association, and self-established enterprises. Self-established enterprises refer to the integration of peach production, procurement, processing, warehousing, logistics, sales, and other links through the establishment of enterprises, to provide integrated services for downstream customers, and not only to improve the industrial chain, but also enhance the product value chain. Therefore, in the questionnaire, we use categorical variables.

#### 3.4.3. Control Variables

Based on the previous studies [42,43], the control variables in this paper are the farmers’ personal characteristics, family operation characteristics, production characteristics, and external environment characteristics.

The definition and assignment of the variables involved in this paper are shown in Table 2.

## 4. Analysis of the Empirical Results

### 4.1. The Influence of Government Regulation and Market Structure on Farmers’ Green Production Behaviors

Due to the possible multicollinearity between the variables, a multicollinearity test was carried out for each variable before the regression. The results show that the VIF was less than 10, indicating that there was no multicollinearity between the variables. In this paper, the ordered probit model was used in Stata15.0. The results are shown in Table 3 below.

#### 4.1.1. The Impact of Government Regulation

The effect of government publicity and training about pesticide application on peach farmers’ behaviors regarding their compliance with pesticide intervals and reading the application instructions during planting, are significant at the level of 1% and 5%, respectively, with a positive coefficient. There was no significant effect on the peach farmers’ application of pesticides in accordance with the standard dosage instructions. During the planting period, if the government supervised and inspected peach farmers’ pesticide application, its influence on the standard dosage of pesticides, whether farmers follow the pesticide interval, and whether they read the pesticide instructions are significant at the level of 1%, 10%, and 1%, respectively, and the coefficients are all positive. This shows that government supervision and inspection of pesticide use promote peach farmers’ green production. By providing biological pesticide subsidies, the government’s influence on farmers’ behaviors, in terms of the standard dosage of pesticides, following the pesticide interval, and reading the pesticide instructions, is significant at the level of 1%, 10%, and 5%, respectively, and the coefficient is positive. This shows that providing biological pesticide subsidies plays a positive role in promoting farmers’ green production behaviors. In summary, government control policies have a positive impact on peach farmers’ green production behaviors. This is because the government can educate peach farmers about green production through measures, such as propaganda and training, supervision and inspection, and biological pesticide control policies. These measures allow peach farmers to correct their bad habits when it comes to applying pesticides, and to start to use biological pesticides, achieving green production.

#### 4.1.2. The Impact of the Market Structure

In general, under the four market structures, establishing self-owned enterprises generally has the greatest impact on peach farmers’ green production behaviors, followed by participating in a peach farmers’ association. In contrast, joining cooperatives and signing contracts with related enterprises have the weakest impact on peach farmers’ green production behaviors.

Specifically, the effects of establishing self-owned enterprises on farmers applying the standard pesticide dosage and reading the application instructions are significant at the level of 10% and 5%, respectively, and the coefficients are positive. The results suggest that the establishment of self-owned enterprises will overall promote peach farmers’ green production performances, because the green production behavior is beneficial to improving both the ecological environment and the peach quality. This helps to increase sales. Therefore, in order to obtain scaled interests, peach farmers having their own enterprises will choose a green production mode. Participating in a peach farmers’ association can significantly improve the peach farmers’ adherence to the recommended pesticide interval at the level of 5%, because a local peach farmers’ association can exert certain constraints on the peach farmers’ production behaviors. However, this informal system has a limited ability to impose constraints, and can only affect the peach farmers’ green production behaviors to a certain extent. Participating in relevant cooperatives and signing contracts with other enterprises have no significant impact on the green production behaviors of peach farmers. Through on-site visits, we found that as the majority of peach farmers’ cooperatives take land for shares, the mechanism of the cooperative members’ benefit connection is loose, and the production management of the cooperatives is decentralized. This leads to a low ability to constrain the peach farmers’ production. When signing a contract with the enterprise, the enterprise is only responsible for collecting the fruit at the picking time and ensuring a sufficient peach supply. Little attention is paid to the production management. Therefore, signing a contract with the enterprise also shows weaker constraints on the peach farmers. These two organizations lead to poor green peach production behaviors.

### 4.2. Testing the Mediation Effect

According to Zhonglin Wen’s idea of testing the mediation effect, we exploit the stepwise regression method in this paper. In the first step, the significance of the total effect between the independent variable X and the dependent variable Y is verified by the OLS model. In the second step, the ordered probit model is employed to test whether the effect of the independent variable X on the mediator variable M is significant. Thirdly, with the control of the independent variable X, we use the OLS model to test whether the effect of the mediator variable M on the dependent variable Y is significant.

The test results are shown in Table 4 and Table 5 below.

Due to the large number of variables, more than one table is required. Therefore, the overall regression of the last step of the mediation effect test is shown separately in Table 5 below.

According to Table 4, the *p* values of the regression coefficient c in model 1 (accounting for the effects of X on Y) demonstrate that government regulation directly and significantly affects the green production behaviors of peach farmers. In model 2, the *p* values of the regression coefficient a (accounting for the effects of X on M) suggest that government regulation has a significant impact on the market structure participation of peach farmers. According to model 3 in Table 5, the *p* values related to c’ and b show that with the influence of government regulation, the market structure, as a mediator variable, still significantly affects the green production behaviors of peach farmers. Here, c’ represents the effects of X on Y with a fixed M, and b characterizes the effects of M on Y with a fixed X. Since the three regression coefficients a, b, and c are all significant, and a, b, and c’ have the same sign, the market structure is shown to have a mediating effect. However, since c’ is also significant, the market structure has a partial but not complete mediation effect, in line with hypothesis 3.

### 4.3. Heterogeneity Test

We will further discuss the green production behaviors of peach farmers from the perspectives of the planting scale and regional heterogeneity.

#### 4.3.1. Differences in the Planting Scale

By understanding the differences in pesticides use between large-scale and small-scale farmers, we can treat different green production behaviors differently and avoid a “one size fits all” policy. A large number of previous studies show that with the expansion of the planting scale, farmers will reduce the pesticide dosage per unit area [44,45]. There also exist contradictory results. As reported by Wang et al. (2017) [46], in the process of rice planting, large-scale farmers apply larger doses of pesticides and do so more often than small-scale farmers. The studied sample in this paper consists of peach farmers. According to the planting scale, peach farmers are divided into large (planting area > 50 Mu) and small scales (planting area < 50 Mu). By comparing the differences in the green production behaviors of the two kinds of peach farmers, we obtain the results shown in Table 6 below.

The above regression results show that in terms of applying the standard pesticide dosage in accordance with the instructions, government supervision and inspection have a significant positive impact on both small-scale and large-scale farmers. Biopesticide subsidies provided by the government play a positive and significant role in the small-scale farmer group but fail the significance test in the large-scale farmer group. However, government publicity and training show a positive and significant effect in the large-scale farmer group, while they fail the significance test in the small-scale farmer group. Thus, it is concluded that publicity and training play a greater role in the large-scale farmer group, while subsidies have a stronger effect in the small-scale farmer group. In terms of the four variables of the organization mode, the participation in cooperatives and self-built enterprises play a significant role in both large-scale and small-scale farmer groups. The regression coefficient of participating in cooperatives in the group of small-scale farmers is larger than that in the large-scale farmer group, while the regression coefficient of self-owned enterprises in the small-scale farmer group is smaller than that of the large-scale farmer group. It can be thus concluded that joining a cooperative encourages more small farmers to apply pesticides in accordance with the prescribed standard dosage, while it is easier for self-owned enterprises to encourage large-scale farmers to apply the standard dosage of pesticides.

In terms of complying with the pesticide interval, only the variable of government publicity and training passed the significance test in both groups of farmers. The magnitude and significance of the coefficients in both groups are almost the same, which suggests that government publicity and training allow all farmers to comply with pesticide intervals. Regarding the variables of the organizational model, the participation in a peach farmers’ association passed the significance test for the group of small-scale farmers, while joining a cooperative passed the significance test for the large-scale farmer group. This indicates that joining a peach farmers’ association encourages small-scale farmers to comply with the pesticide interval, while joining a cooperative is more effective for large-scale farmers.

In terms of reading the instructions for pesticide use, the two government-regulated variables of “government publicity and training” and “government supervision” passed the significance test in the small-scale farmer group, while the third variable, “government biopesticide subsidies”, passed the significance test in the large-scale farmer group. Therefore, government publicity, training, and supervision are more likely to encourage small-scale farmers to read the pesticide instructions, while government subsidies for biopesticides are more likely to encourage large-scale farmers to read the pesticide instructions. For the four variables of the organizational model, participating in cooperatives passed the significance test in both groups of large- and small-scale farmers, while the other variables were not significant in these two farmer groups. This shows that joining a cooperative helps all farmers to read the pesticide instructions, so as to regulate their own production behaviors.

#### 4.3.2. Area Differences

The local economic level has an impact on the green production behavior of farmers [47]. Therefore, in this paper, according to the level of the regional economy in China, the sample of peach farmers was divided into three groups by region: the east, the middle, and the west. The regression analysis was then carried out, and the results are shown in Table 7.

The above regression results show that, firstly, in terms of “whether peach farmers apply the standard dosage pesticide”, the two government-regulated variables of “supervision and inspection” and “biopesticide subsidy” passed the significance test in the eastern and western regions, both showing a similar significance. Only the variable of “supervision and inspection” passed the significance test in the middle region. The variable of “government publicity and training” failed the significance test in all three regions. For the four variables relating to the organization mode, the variable of self-owned enterprises passed the significance test in the eastern region, and in the western region, the mode of signing contracts with other enterprises passed, while in the central region, none of the organizational modes passed the significance test.

Secondly, in terms of “complying with the pesticide interval”, government publicity and training will have a positive and significant effect on peach farmers in the central and eastern regions. For peach farmers in the western region, government supervision and inspection and biological pesticide subsidies play a positive role in promoting the appropriate application intervals. For the organization modes, self-owned enterprises encourage peach farmers in the eastern region to comply with the pesticide interval; for peach farmers in the central region, joining the peach farmers’ association has a positive effect on complying with the pesticide interval; and for peach farmers in the western region, joining a cooperative and contracting with others encourage this behavior.

Thirdly, in terms of “whether reading instructions for the pesticide use”, both “government supervision” and “government biopesticide subsidies” have a positive and significant impact on peach farmers in the eastern and western regions, while the variable of “government publicity and training” plays a positive role in encouraging peach farmers in the central region to read pesticide instructions. In terms of the four variables relating to the organization mode, self-owned enterprises play a greater role in the standardization of peach pesticides in the eastern region. Joining an association helps peach farmers in the western region to read the pesticide instructions, while for peach farmers in the middle region, none of the four organization modes passed the significance test.

### 4.4. Robust Test

In this paper, the robust test is carried out via the substitution statistics method. We use the Logit model to test the robustness of the factors that affect the green production behavior of the sample farmers. The results are shown in Table 8.

In Table 8, it can be seen that the significance and sign of the core variables do not vary much, indicating that the benchmark results are robust.

## 5. Discussion

This paper finds that government regulation and market structure can indeed promote farmers to implement a green production behavior, which is basically consistent with the existing research conclusions [48,49], and also confirms the reliability of this study. However, different from the existing research, this paper focuses on discussing the specific impact of three types of government regulation and four types of market structure on the green production behavior of farmers, which enriches the existing theory regarding farmers’ behaviors and provides new ideas for promoting the green development of agriculture, which has a certain theoretical and practical significance.

Firstly, from the perspective of government regulation, it can be seen from Table 3 that guidance regulation, incentive regulation, and constraint regulation all have a certain impact on the green production behaviors of farmers. Once farmers are found to have used highly toxic banned pesticides or to have made excessive use of pesticides, strict punishment and regulatory measures will bring them additional burden. The green production subsidies given by the government can, to a certain extent, compensate for the positive externalities brought about by the green production of farmers, thus increasing the income of farmers. This is consistent with previous research conclusions [50]. Therefore, from the perspective of the “rational economic man”, farmers will comply with the safety production standards and apply pesticides as required. Based on this, we believe that the government can adopt the policy combination of “carrot and stick” to encourage farmers to conduct green production and promote the agricultural transformation and upgrading [4].

Secondly, from the perspective of the market structure, it can be seen from Table 4 and Table 5 that a sound market structure can promote the implementation of a green production behavior. This is because the establishment of a relationship of interest between farmers and industrial organizations, leading to the formation of a stable market structure, can continue to increase farmers’ incomes, and thus force farmers to carry out green production [51]. On the one hand, under a sound market mechanism, information between the buyer and the seller is equal, allowing quality grading and product elimination, which means that farmers who do not engage in green production will face an excess profit loss. Therefore, in order to pursue profits, farmers will use pesticides reasonably and safely. On the other hand, by joining the industrial organization, farmers link their own interests with the industrial organization, engage in agricultural production in accordance with the contract, and accept the agricultural materials and technical guidance provided by the industrial organization in the production process, based on the premise of obtaining a guaranteed benefit. To a certain extent, farmers are included in the green production reform [49].

Finally, this paper also analyzed the heterogeneity from the two perspectives of planting scale and region. According to Table 6, government supervision, inspection, and subsidies play a greater role for small farmers, while government publicity and training have a greater impact on large-scale farmers. This may be because small and large farmers have different responses to different control methods [52,53]. Small farmers need certain regulatory means and subsidies to encourage them to carry out green production because of their dispersion. However, large farmers need more knowledge of green production in order to further apply it to production. Participation in industrial organizations has a greater impact on large-scale farmers. This may be because there is a certain threshold for the access of some market structures. Therefore, the current market structure is not fully able to drive small farmers to green production and management. According to Table 7, the impact of government regulation measures and market structure in different regions is significantly different, which is closely related to the economic development of each region [48]. In the east, government supervision and inspection play a greater role; in the middle, government publicity and training play a greater role; and in the west, government subsidies play a greater role. Similarly, participation in cooperatives and signing contracts with enterprises play a greater role in driving farmers in the western region; participation in peach farmers’ associations has the greatest impact on farmers in the central region; and farmers in the eastern region rely more on self-built enterprises to achieve a green production.

## 6. Conclusions and Policy Suggestions

### 6.1. Conclusions

Government regulations and market structures have a positive and significant impact on the green production of peach farmers. Government regulations, consisting of publicity, training, supervision, inspection, and subsidies for biological pesticides, all have a positive and significant impact on the green production behavior of peach farmers. In the market structure, establishing self-owned enterprises and participating in peach farmers’ associations have a positive and significant impact on the green production behaviors of peach farmers.

Government-regulated publicity and training do not pass the significance test of applying pesticides in accordance with the standard dosage in the manual—one of the green production behaviors of peach farmers. This suggests that there is a phenomenon of “relative system failure” in the propaganda and training activities organized by the government during the peach planting period. Joining cooperatives and signing contracts with other enterprises in the market structure also failed the significance test with regard to peach farmers’ green production behaviors. This shows that not all market structures can promote the green production behavior of peach farmers, and some of them place insufficient constraints on green production behavior.

Government regulation can affect peach farmers’ green production behaviors through the market structure. The market structure plays a partial intermediary role in the way government regulation affects peach farmers’ green production behavior.

### 6.2. Policy Suggestions

With these conclusions, we make the following policy suggestions. (1) Relevant policies should be formulated according to the local conditions in different regions, focusing on different peach farmers at different scales. In general, the government should actively promote the publicity and training of green agricultural production, supervise the implementation of government subsidies, and promote the green production of farmers. At the same time, we should also strengthen the supervision and inspection of peach farmers in the process of planting, and improve the compliance of peach farmers with policies. That is, the government should “give them a leg up to get them going”, so as to ensure the stable and lasting implementation of a green production behavior. (2) The market structure plays an important role in encouraging farmers to implement a green production behavior. Therefore, we should optimize the fruit market environment, improve the market structure, considering the market structure as an important way to guide farmers to implement a green production, and strengthen the binding force of the market structure. We should promote the healthy, standardized, and orderly development of farmers’ cooperatives, family farms, agricultural industrialization consortia, and other new agricultural business entities, guide new agricultural business entities to enhance their key development capabilities, stimulate their internal vitality, carry out intensive and standardized productions, improve the benefit sharing mechanism, and better play a leading role in driving small farmers to enter the market, increase income, and build modern agriculture. At the same time, we should carry out quality inspection and product traceability for agricultural products, strengthen the premium purchase of high-quality agricultural products by enterprises, cooperatives, and other entities, improve the mechanisms that lead to high quality and good prices, resolutely prevent the “bad money driving out good” phenomenon, and improve the endogenous motivation of farmers for green production. (3) Based on Ostrom’s theory of polycentric governance and the conclusions drawn in this paper, it is argued that a single model of government or market is prone to huge transaction costs, a low efficiency, and poor governance continuity. Therefore, in order to guide the green production behavior of peach farmers, the government and the market should draw clear guidelines, by which the government handles the government’s business, and the market handles the market’s business. This improves the work efficiency of the government and the market, and also helps to maintain the stability of the green production behavior of peach farmers.

### 6.3. Limitations and Prospects

The limitations of this study include the following two aspects: on the one hand, the data used in this study cannot dynamically reflect the impact of government regulation and market structure on the green production behaviors of farmers. Because it is difficult to obtain dynamic panel data, only static cross-sectional data can be used for the correlation analysis, which prevents the research results from being able to reflect the impact of government regulation on farmers’ green production behaviors in different stages, as well as the impact of changes in the market structure in different periods on farmers’ safe pesticide use. On the other hand, as with most previous studies, due to the limitations in terms of funds and time, we used the questionnaire method to obtain the data needed for this study; that is, the data used were self-reported by farmers. However, due to the limited level of education and subjective awareness of farmers, the data may exhibit some deviation. In future research, we can use the quasi-natural experiment method to observe and record the green production behaviors of farmers, and track and investigate the sample farmers to obtain more accurate panel data for verification.

## Figures and Tables

**Table 1:** Descriptive statistics.

Variables	Group	Number of Farmers	Proportion
Gender	Male	676	91.23%
	Female	65	8.77%
Educational level	Primary school and below	59	7.96%
	Junior high school	318	42.91%
	High school	248	33.47%
	Junior college	90	12.15%
	University and above	26	3.51%
Planting years	≤10 years	379	51.15%
	10–20 years	243	32.79%
	≥20 years	119	16.06%
Planting scale	≤50 Mu	517	69.77%
	50–100 Mu	73	9.85%
	100–1000 Mu	146	19.71%
	≥1000 Mu	5	0.67%

**Table 2:** Model variable assignment and the descriptive statistics.

Variable Category	Variable Title	Variable Assignment	Mean	Min	Max
Dependent variables	Pesticides are applied in accordance with the specified dosage instructions	1 = not at all; 2 = usually not; 3 = neutral; 4 = generally yes; 5 = every time	4.147	1	5
	The pesticide interval is followed	1 = completely non-compliance; 2 = generally non-compliance; 3 = neutral; 4 = generally compliance; 5= complete compliance	4.544	1	5
	Farmers read the instructions	1 = not reading; 2 = do not usually read; 3 = neutral; 4= usually read; 5= every time	4.451	1	5
Independent variables	The government has conducted publicity and training on pesticide use during planting	0 = no; 1 = yes	0.780	0	1
	The government has conducted supervision and inspection of pesticide use during planting	0 = no; 1 = yes	0.699	0	1
	The government has provided biological pesticide subsidies	0 = no; 1 = yes	0.158	0	1
	Main organizational models	0 = none; 1 = cooperatives; 2= contract farming; 3 = self-established enterprises; 4 = joining a peach farmers association	1.247	0	4
Control variables	Gender	1 = male; 0 = female	0.912	0	1
	Education level	1 = primary school and below; 2 = junior high school; 3 = high school; 4 = junior college; 5 = university and above	2.603	1	5
	Business entity	0 = small farmers; 1 = professional large households; 2 = cooperatives; 3 = enterprises; 4 = research institutes	2.212	1	5
	Management mode	1 = completely independent management; 2 = hire technical staff; 3 = independent management and hire technical staff	1.309	1	3
	Type of peach orchard	1 = open-field peach; 2 = facility peach; 3 = open-field peach and facility peach	1.165	1	3
	Planting year	( ) Year	13.157	0	50
	Total area of peach orchard	( ) Mu (1 Mu = 0.16 acres)	89.383	0.8	3700
	Have “San pin yi biao” certification	0 = none; 1 = pollution-free; 2= green; 3 = organic; 4 = geographical indication products (According to the current domestic and international standards, the research group divides agricultural products into the following four categories in the questionnaire, namely, pollution-free, green, organic, and national geographical indication products)	0.877	0	4
	Have applied for a registered trademark	0 = no; 1 = yes	0.324	0	1
	Terrain of peach orchard	1 = plain; 2= hills; 3 = mountain; 4 = plateau	1.857	1	4

**Table 3:** Regression results of the benchmark model.

Variable		Model 1	Model 2	Model 3
		Whether Pesticides Are Applied in Accordance with the Specified Dosage Instructions	Whether the Pesticide Interval Is Followed	Whether Farmers Read the Instructions
Government regulation	Government has conducted publicity and training	0.067	0.420 ***	0.347 **
		(0.50)	(2.91)	(2.51)
	Government has conducted supervision and inspection	0.482 ***	0.248 *	0.400 ***
		(3.85)	(1.84)	(3.10)
	Government has provided biological pesticide subsidies	0.353 ***	0.267 *	0.302 **
		(2.65)	(1.85)	(2.12)
Market structure	Joining cooperatives	0.108	−0.091	0.008
		(0.96)	(−0.75)	(0.06)
	Contract farming	0.072	0.050	0.147
		(0.34)	(0.22)	(0.67)
	Self-owned enterprises	0.312 *	0.232	0.429 **
		(1.80)	(1.22)	(2.27)
	Joining a peach farmers association	0.229	0.425**	0.197
		(1.13)	(2.03)	(0.95)
Control variables	Gender	0.154	0.293	0.073
		(1.03)	(1.88)	(0.47)
	Business entity	0.109 ***	0.104 ***	0.103 ***
		(3.88)	(3.47)	(3.52)
	Management mode	0.195 ***	0.116	0.027
		(2.81)	(1.58)	(0.38)
	Type of peach orchard	0.102	−0.086	0.110
		(1.11)	(−0.90)	(1.12)
	Planting year	0.008	−0.004	0.001
		(1.60)	(−0.75)	(0.22)
	Educational level	0.010	0.147 ***	0.097
		(0.21)	(2.72)	(1.84)
	Planting scale	−0.000	0.000	−0.000
		(−0.73)	(0.35)	(−0.47)
	Have “San pin yi biao” certification	0.083 **	0.011	0.072
		(2.11)	(0.26)	(1.75)
	Have applied for a registered trademark	0.058	0.148	0.025
		(0.54)	(1.29)	(0.22)
	Terrain of peach orchard	0.056	0.094	−0.078
		(1.04)	(1.61)	(−1.38)

Annotation: ***, **, and * represent the significance level at 1%, 5%, and 10%, respectively; t statistics are given in parentheses.

**Table 4:** Testing the mediation effect.

	Model 1			Model 2			
Model Type	OLS			Ordered Probit			
Dependent variables	Whether pesticides are applied in accordance with the specified dosage instructions	Whether the pesticide interval is followed	Whether farmers read the instructions	Joining cooperatives	Contract farming	Self-owned enterprises	Joining a peach farmers’ association
Government has conducted publicity and training	0.033 (0.24)	0.248 *** (3.43)	0.272 *** (2.98)	0.047 (0.18)	0.717 * (1.88)	0.010 (0.03)	0.220 (0.54)
Government has conducted supervision and inspection	0.514 *** (4.12)	0.119 * (1.79)	0.224 *** (2.66)	0.232 (1.00)	0.675 * (1.88)	0.771 ** (2.35)	0.861 ** (2.25)
Government has provided biological pesticide subsidies	0.267 ** (2.16)	0.078 (1.18)	0.169 ** (2.04)	0.583 ** (2.05)	0.651 * (1.77)	0.500 (1.52)	0.111 (0.29)
Gender	0.153 (1.04)	0.159 ** (2.02)	0.021 (0.21)	−0.236 (−0.85)	−0.449 (−1.19)	0.447 (1.00)	−0.427 (−1.14)
Business entity	0.119 *** (4.31)	0.043 *** (2.91)	0.068 *** (3.66)	0.075 (1.51)	0.071 (0.89)	0.185 *** (2.91)	0.140 (1.64)
Business mode	0.167 *** (2.60)	0.054 (1.58)	0.025 (0.59)	0.666 *** (2.69)	0.717 *** (2.64)	0.802 *** (3.08)	0.559** (2.03)
Type of peach orchard	0.125 (1.46)	−0.050 (−1.09)	0.069 (1.21)	0.198 (0.90)	0.207 (0.73)	0.542 ** (2.28)	0.234 (0.87)
Planting year	0.006 (1.18)	−0.002 (−0.70)	−0.000 (−0.11)	−0.006 (−0.60)	−0.032 ** (−2.23)	−0.013 (−1.07)	−0.047 *** (−3.30)
Educational level	0.002 (0.05)	0.064 ** (2.51)	0.053 (1.65)	0.362 *** (3.49)	0.349 ** (2.51)	0.490 *** (3.92)	0.480 *** (3.50)
Planting scale	−0.000 (−1.62)	0.000 (0.20)	−0.000 (−0.52)	0.007 *** (3.56)	0.008 *** (3.71)	0.008 *** (3.67)	0.008 *** (3.71)
Have “San pin yi biao” certification	0.104 *** (2.70)	0.006 (0.29)	0.064 ** (2.44)	0.048 (0.59)	0.106 (0.96)	0.005 (0.05)	0.122 (1.16)
Have applied for a registered trademark	0.029 (0.28)	0.072 (1.32)	0.038 (0.56)	1.284 *** (5.03)	0.894 *** (2.75)	1.324 *** (4.46)	1.398 *** (4.54)
Terrain of peach orchard	0.039 (0.74)	0.045 (1.64)	0.054 ( 1.55)	0.480 *** (4.67)	0.586 *** (3.99)	0.493 *** (3.63)	0.449 *** (2.98)

Annotation: ***, **, and * represent the significance level at 1%, 5%, and 10%, respectively; t statistics are given in parentheses.

**Table 5:** Testing the mediation effect.

Model 3	OLS		
Dependent variables	Whether pesticides are applied in accordance with the specified dosage instructions	Whether the pesticide interval is followed	Whether farmers read the instructions
Government has conducted publicity and training	0.037 (0.27)	0.249 *** (3.43)	0.279 *** (3.04)
Government has conducted supervision and inspection	0.497 *** (3.95)	0.120 * (1.79)	0.214 ** (2.53)
Government has provided biological pesticide subsidies	0.269 ** (2.17)	0.075 (1.14)	0.165 ** (1.98)
Joining cooperatives	0.052 (0.45)	0.054 (0.89)	0.027 (0.35)
Contract farming	0.043 (0.21)	0.011 (0.10)	0.108 (0.78)
Self-owned enterprises	0.222 (1.34)	0.059 (0.67)	0.159 (1.43)
Joining a peach farmers’ association	0.201 (1.02)	0.199 * (1.89)	0.108 (0.81)
Gender	0.143 (0.97)	0.149 * (1.90)	0.011 (0.11)
Business entity	−0.122 *** (−4.34)	−0.049 *** (−3.25)	−0.075 *** (−3.95)
Business mode	0.160 ** (2.47)	0.051 (1.48)	0.018 (0.41)
Type of peach orchard	0.111 (1.30)	−0.057 (−1.25)	0.056 (0.98)
Planting year	0.006 (1.26)	−0.002 (−0.87)	−0.000 (−0.12)
Educational level	−0.008 (−0.15)	0.066 ** (2.56)	0.050 (1.55)
Planting scale	−0.000 * (−1.70)	0.000 (0.24)	−0.000 (−0.58)
Have “San pin yi biao” certification	−0.104 *** (−2.68)	0.008 (0.38)	−0.062 ** (−2.40)
Have applied for a registered trademark	0.005 (0.05)	−0.059 (−1.06)	0.042 (0.60)
Terrain of peach orchard	0.033 (0.62)	0.048 * (1.69)	−0.057 (−1.60)

Annotation: ***, **, and * represent the significance level at 1%, 5%, and 10%, respectively; t statistics are given in parentheses.

**Table 6:** Model regression results of the peach farmers with different planting sizes.

	Whether Pesticides are Applied in Accordance with the Specified Dosage Instructions		Whether the Pesticide Interval is Followed		Whether Farmers Read the Instructions	
	small-scale	large-scale	small-scale	large-scale	small-scale	large-scale
Government has conducted publicity and training	−0.154 (−0.92)	0.701 *** (2.79)	0.559 *** (3.10)	0.601 ** (2.23)	0.359 ** (2.10)	0.542 ** (2.13)
Government has conducted supervision and inspection	0.539 *** (3.62)	0.481 ** (1.89)	0.139 (0.86)	0.416 (1.56)	0.433 *** (2.81)	0.315 (1.22)
Government has provided biological pesticide subsidies	0.629 *** (3.48)	−0.113 (−0.51)	0.236 (1.27)	0.333 (1.27)	0.171 (0.91)	0.440 ** (1.82)
Joining cooperatives	0.215 * (1.72)	1.050 ** (2.34)	0.037 (0.28)	0.744 * (1.80)	0.107 (0.82)	0.887 ** (2.07)
Contract farming	−0.262 (−0.88)	0.728 (1.44)	−0.234 (−0.74)	0.180 (0.37)	−0.070 (−0.22)	0.482 (0.99)
Self-owned enterprises	0.401 * (1.76)	0.832 ** (1.79)	0.328 (1.31)	0.211 (0.48)	0.549 ** (2.12)	0.391 (0.86)
Joining a peach farmers’ association	0.012 (0.04)	0.207 (0.38)	0.647 ** (2.27)	0.713 (1.42)	−0.676 ** (−2.47)	0.261 (0.50)
Control variable	Controlled					
N	498	243	498	243	498	243

Annotation: ***, **, and * represent the significance level at 1%, 5%, and 10%, respectively; z statistics are given in parentheses.

**Table 7:** The regression results of the peach farmer model in the eastern, central, and western regions.

	Whether Pesticides are Applied in Accordance with the Specified Dosage Instructions			Whether the Pesticide Interval is Followed			Whether Farmers Read the Instructions		
	Eastern	Central	Western	Eastern	Central	Western	Eastern	Central	Western
Government has conducted publicity and training	0.025 (0.13)	0.006 (0.02)	−0.048 (−0.15)	0.409 * (1.95)	0.812 *** (2.62)	−0.061 (−0.18)	0.058 (0.29)	1.226 *** (4.18)	0.140 (0.43)
Government has conducted supervision and inspection	0.334 * (1.88)	0.674 ** (2.40)	0.522 * (1.83)	0.182 (0.97)	0.049 (0.15)	0.588 * (1.86)	0.364 ** (2.01)	0.158 (0.54)	0.583 * (1.95)
Government has provided biological pesticide subsidies	0.411 ** (2.33)	0.143 (0.25)	0.585 ** (2.11)	0.159 (0.84)	−0.135 (−0.20)	0.842 ** (2.43)	0.400 ** (2.11)	0.757 (1.01)	0.627 ** (2.05)
Joining cooperatives	0.248 (1.54)	−0.085 (−0.28)	0.225 (1.02)	−0.034 (−0.20)	−0.136 (−0.41)	0.590 ** (2.28)	−0.005 (−0.03)	−0.147 (−0.45)	0.087 (0.37)
Contract farming	−0.088 (−0.32)	−0.321 (−0.49)	0.829 * (1.82)	0.345 (1.16)	0.598 (0.80)	0.997 ** (2.20)	0.382 (1.32)	1.064 (1.28)	−0.274 (−0.63)
Self-owned enterprises	0.548 ** (2.38)	−0.097 (−0.23)	0.251 (0.59)	0.444 * (1.81)	−0.090 (−0.20)	0.142 (0.26)	0.654 *** (2.59)	0.331 (0.70)	0.287 (0.59)
Joining a peach farmers’ association	0.261 (0.97)	0.654 (1.20)	−0.623 (−1.27)	0.173 (0.59)	1.594 *** (3.04)	1.394 ** (2.45)	0.268 (0.93)	−0.726 (−1.42)	1.547 *** (3.04)
Control variable	Controlled								
N	379	152	210	379	152	210	379	152	210

Annotation: ***, **, and * represent the significance level at 1%, 5%, and 10%, respectively; z statistics are given in parentheses.

**Table 8:** Robustness test.

	Whether Pesticides Are Applied in Accordance with the Specified Dosage Instructions	Whether the Pesticide Interval Is Followed	Whether Farmers Read the Instructions
Government has conducted publicity and training	0.153 (0.67)	0.661 *** (2.68)	0.582 ** (2.49)
Government has conducted supervision and inspection	0.808 *** (3.74)	0.426 * (1.86)	0.687 *** (3.15)
Government has provided biological pesticide subsidies	0.656 *** (2.80)	0.654 ** (2.52)	0.493 ** (1.98)
Joining cooperatives	0.214 (1.14)	−0.115 (−0.55)	0.066 (0.33)
Contract farming	0.218 (0.60)	0.135 (0.34)	0.313 (0.83)
Self-owned enterprises	0.618 ** (2.09)	0.569 * (1.70)	0.949 *** (2.80)
Joining a peach farmers’ association	0.366 (1.07)	0.736 ** (2.08)	0.265 (0.76)
Gender	0.219 (0.86)	0.454 * (1.70)	0.137 (0.51)
Business entity	−0.201 *** (−4.24)	−0.190 *** (−3.74)	−0.173 *** (−3.41)
Business mode	0.356 *** (2.91)	0.246 * (1.87)	0.061 (0.49)
Type of peach orchard	0.162 (1.03)	−0.043 (−0.25)	0.186 (1.06)
Planting year	0.015 * (1.73)	−0.005 (−0.56)	0.005 (0.54)
Educational level	0.038 (0.44)	0.258 *** (2.74)	0.184 ** (1.98)
Planting scale	−0.000 (−0.61)	0.000 (0.42)	−0.000 (−0.22)
Have “San pin yi biao” certification	−0.142 ** (−2.13)	0.002 (0.03)	−0.113 (−1.56)
Have applied for a registered trademark	−0.162 (−0.89)	−0.352 * (−1.79)	−0.036 (−0.19)
Terrain of peach orchard	0.116 (1.27)	0.161 (1.60)	−0.147 (−1.51)

Annotation: ***, **, and * represent the significance level at 1%, 5%, and 10%, respectively; z statistics are given in parentheses.

## Data Availability

The associated dataset of the study is available upon request to the corresponding author.

## References

[1] Statistics Bureau of the People’s Republic of China (2021). China Statistical Yearbook.

[2] He Y., Qi Y. (2021). An Empirical Study on the Formation Mechanism of Farmers’ Green Production Behavior: Based on the Investigation of Fertilization Behavior of 860 Citrus Growers in Sichuan and Chongqing. Resour. Environ. Yangtze Basin.

[3] Feng Y., Geng Y., Liang Z., Shen Q., Xia X. (2021). Research on the Impacts of Heterogeneous Environmental Regulations on Green Productivity in China: The Moderating Roles of Technical Change and Efficiency Change. Int. J. Environ. Res. Public Health.

[4] Xu Y., Liang J., Dong Z., Shi M. (2022). Can Environmental Regulation Promote Green Innovation and Productivity? The Moderating Role of Government Interventions in Urban China. Int. J. Environ. Res. Public Health.

[5] Luo X., Du S., Huang Y., Tang L., Yu W. (2020). Planting Scale, Market Regulation and Rice Farmers’ Biological Pesticide Application Behavior. J. Agrotech. Econ..

[6] Shi Z., Zhang K. (2021). Research on farmers’ adoption behavior of green prevention and control technology. J. Arid Land Resour. Environ..

[7] Huang Z., Zhong Y., Wang X. (2016). Study on the impacts of government policy on farmers’ pesticide application behavior. China Popul. Resour. Environ..

[8] Yang C.F., Heng Y.Z., Zhang Y.F. (2022). Are Socialized Services of Agricultural Green Production Conducive to the Reduction in Fertilizer Input? Empirical Evidence from Rural China. Int. J. Environ. Res. Public Health.

[9] Zhu W., Wang R.H. (2022). Land management scale, number of plots, land transfer and pesticide reduction. Chin. J. Agric. Resour. Reg. Plan..

[10] Jin Z.W. (2020). Study on Pesticide Reduction Based on the Scale of Food and Agriculture Land Management—Taking Xihua County as an Example. Master’s Thesis.

[11] Ma W., Abdulai A., Goetz R. (2018). Agricultural cooperatives and investment in organic soil amendments and chemical fertilizer in China. Am. J. Agric. Econ..

[12] Wang B., Huang D. (2005). The Theory of Government Regulation under the Paradigm of “Market Structure Market Behavior Market Performance” and Its Reference to China. Shandong Soc. Sci..

[13] Bambio Y., Agha S.B. (2018). Land tenure security and investment: Does strength of land right really matter in rural Burkina Faso?. World Dev..

[14] Huang J. (2020). A Study on the Influence of Cooperatives on the Adoption Behavior of Green Agricultural Production Technology among Rice Farmers. Master’s Thesis.

[15] Zhao X., Zheng J., Zhang M. (2020). Analysis of green production behavior in the “tea farmer planting cooperative” model based on the principal agent theory. World Agric..

[16] Luo L., Qiao D., Zhang R., Luo C., Fu X., Liu Y. (2022). Research on the Influence of Education of Farmers’ Cooperatives on the Adoption of Green Prevention and Control Technologies by Members: Evidence from Rural China. Int. J. Environ. Res. Public Health.

[17] Zhu P., Zheng J., Zhang M., Zhao X. (2022). Can Participation in Cooperatives Promote the Application Behavior of Green Planting Technology by Grain Farmers?—Based on the Perspective of Endogenous Motivation and External Constraints. World Agric..

[18] Marenya P., Barrett C. (2009). Soil quality and fertilizer use rates among small holder farmers in Western Kenya. Agric. Econ..

[19] Nie Z.F. (2022). Research on the Impact of Rurale-commerce on Rural Economic Transformation—Take the Four Provinces in Central China As. Master’s Thesis.

[20] Hu R., Yang Z., Kelly P., Huang J. (2009). Agricultural extension system reform and agent time allocation in China. China Econ. Rev..

[21] Chu C., Feng S., Zhang W. (2012). An empirical analysis of farmers’ adoption of environment-friendly agricultural technologies: A case study of organic fertilizer and soil testing formula fertilization technology. Chin. Rural. Econ..

[22] Liu H., Han X.Y., Xue Y., Piao H.L., Lv J. (2022). Influence of social network and environmental literacy on farmers’ excessive application of chemical fertilizer: Based on survey data from maize farmers of the three provinces in Northeast China. J. China Agric. Univ..

[23] Wang L., Tang J., Tang M., Su M., Guo L. (2022). Scale of Operation, Financial Support, and Agricultural Green. Int. J. Environ. Res. Public Health.

[24] Zheng Y. (2022). Study on Farmers’ Green Production Technology Adoption Behavior and Its Influencing Factors. Master’s Thesis.

[25] Lv X., Li D., Zhou H. (2018). Discussion on Quality and Safety of Agricultural Products: Concurrent Business and Pesticide Application Behavior--Evidence from Hunan, Jiangxi and Jiangsu Provinces. China Agric. Univ. J. Soc. Sci. Ed..

[26] Luo L., Liu Y.C., Wu X.Y., Wang Y.N. (2022). The path of individual and situational factors to activating farmers’ green production. J. Hunan Agric. Univ. (Soc. Sci.).

[27] Damalas C.A. (2021). Farmers’ intention to reduce pesticide use: The role of perceived risk of loss in the model of the planned behavior theory. Environ. Sci. Pollut. Res. Int..

[28] Sun X., Lyu J., Ge C. (2022). Knowledge and Farmers’ Adoption of Green Production Technologies: An Empirical Study on IPM Adoption Intention in Major Indica-Rice-Producing Areas in the Anhui Province of China. Int. J. Environ. Res. Public Health.

[29] Stigler G.J. (2021). The theory of economic regulation. The Political Economy Readings in the Politics and Economics of American Public Policy.

[30] Spulber D.F. (2003). Regulation and Markets.

[31] Gilbert (2004). The way of industrial supervision and its political economy. Compare.

[32] Borland J., Yang X. (1992). Specialization and a new approach to economic organization and growth. Am. Econ. Rev..

[33] Swedberg R. (2005). Principles of Economic Sociology.

[34] Zhong Y.Q., Huang Z.H., Wu L. (2016). Difference Between Willingness and Behavior of Farmers’ Participation in Cooperatives: An Empirical Analysis and Policy Proposal. J. Northwest AF Univ. Soc. Sci. Ed..

[35] Jiang Y., Gao J. (2013). Rural Household Behaviors and Quality Safety of Agricultural Products in Different Organization Structures of Agricultural Operations. J. Yunnan Univ. Financ. Econ..

[36] Zhang M. (2010). Organization Model of Agricultural Products Supply Chain and Agricultural Products Quality and Safety. Rural. Econ..

[37] Wen Z., Zhang L., Hou J., Liu H. (2004). Testing and Application of the Mediating Effects. Acta Psychol. Sin..

[38] Li F., Zhang J., He K. (2019). Alternative and Complementary: Informal Institutions and Formal Institutions in Farmers’ Green Production. J. Huazhong Univ. Sci. Technol. Soc. Sci. Ed..

[39] Wang Y., Xu X., Qiu F. (2017). Current Status of Tea Green Production and Suggestions of the Sustainable Development of Tea Industry. Hubei Agric. Sci..

[40] Zhang H.L., Li J.Y., Teng H.Q. (2020). Cognition, external environment and green agricultural technology adoption behavior for small-scale farmers. J. Arid Land Resour. Environ..

[41] Geng Y.N., Zheng S.F., Wang J.H. (2017). Impact of the Government Technology Promotion and Supply Chain Organization on Farmers’ Biological Technology Adoption Behavior. J. Northwest AF Univ. Soc. Sci. Ed..

[42] Qin S. (2020). Studies on the Spraying Behavior of Rice Farmers. Doctoral Dissertation.

[43] Wu X. (2016). A Study on Farmers’ Green Agricultural Technology Adoption Behavior and Policy Incentives. Ph.D. Thesis.

[44] Gong J., Huang M., Ma Y., Sun J. (2016). Cultural Background, Eco-Environment Awareness and Pesticide Application Behavior of Farmers. J. Ecol. Rural Environ..

[45] Du S., Zheng J.F. (2021). Analysis on the Pesticide Over-application Behavior of Wheat Farmers Based on Logit-ISM Model. J. Anhui Agric. Sci..

[46] Wang M., Liu Y., Gao Q., Liu D. (2017). The Spatial and Temporal Analysis of the Comparative Advantage of different Rice Planting Pattern in Hubei Province. Econ. Geogr..

[47] Zhang J., Zhang K., Zhang G., Guo L. (2021). Family Farm Green Production Behavior Choice and Regional Comparative Study. Agric. Econ. Manag..

[48] Xu L., Li H. (2022). Governmental Regulations, Community Actions and the Sustainable Level of Green Production of Tea Farmers. Issues For. Econ..

[49] Zhang K.J., Yu F.W., Yin C.B. (2021). An Mechanism Analysis of the Influence of Industrial Organization Mode on Rice Farmers’ Green Production Behaviors. Rural Econ..

[50] Qiao D.K., Luo L., Zheng X.Q., Fu X.H. (2022). External Supervision, Face Consciousness, and Pesticide Safety Use: Evidence from Sichuan Province, China. Int. J. Environ. Res. Public Health.

[51] Yuan X.P., Liu T.J., Hou X.K. (2019). The Impact of Transaction Mode on Growers’ Safe Production Behavior—Empirical Analysis of 1001 Growers from Main Apple-producing Areas. J. Agrotech. Econ..

[52] Yu Y., Liu X.H., Song Y., Wu Y.P. (2021). Government Regulation, Dual Embedded Governance, Green and Healthy Breeding Behavior—Empirical Analysis Based on Survey Data of Henan Province. J. Agrotech. Econ..

[53] He Y. (2019). Research on the Formation Mechanism and Realization Path of Peasants’ Green Production Behavior—Based on the Evidence of Chemical Inputs from Citrus Growers. Ph.D. Thesis.

